# Branch Waveguide Couplers with a frequency of 510 GHz for Terahertz Transmit/Receive Isolation Applications

**DOI:** 10.3390/mi15091083

**Published:** 2024-08-28

**Authors:** Hao Li, Dehai Zhang, Jin Meng, Li Wang

**Affiliations:** 1 University of Chinese Academy of Sciences, Beijing 100049, China; 2 The CAS Key Laboratory of Microwave Remote Sensing, National Space Science Center, Chinese Academy of Sciences, Beijing 100190, China

**Keywords:** waveguide coupler, terahertz circuit, submillimeter−wave device, multi−branch coupler

## Abstract

To address the requirement of functioning as a transmit/receive isolation device in terahertz transceiver systems, in this paper, we present two high−isolation multi−branch waveguide directional couplers operating at a center frequency of 510 GHz. One is a high−performance five−branch directional coupler, and the other is a new type of three−branch waveguide coupler with lower processing difficulty. Both couplers were fabricated using low−cost CNC milling technologies. The performance of these couplers was verified through measurement results, demonstrating high isolation at the center frequency.

## 1. Introduction

The terahertz waves (THz) usually refer to electromagnetic waves with frequencies ranging from 0.1 to 10 THz. Due to its special situation between microwave and infrared frequencies, THz technology is of great importance for academic and engineering research in various fields, such as biomedical science, space research, astrophysics, and wireless communication [1].

With the swift progress of THz technology, dependable and high−performance THz transceiver systems have become research hotspots. In terahertz systems, achieving a high level of isolation between the transmission and reception paths has substantial significance in optimizing system sensitivity and signal−to−noise ratios. Nevertheless, the implementation of transmit/receive isolation devices at frequencies of 500 GHz and beyond is a major challenge.

There are several approaches to achieving initial isolation in terahertz systems through hardware. One method is to use two sets of antennas to provide spatial isolation; however, this significantly increases the system’s size and complexity. Another approach is to use switches to alternate the primary links. However, terahertz switches operating at 500 GHz and higher frequencies are not commonly available as their size, speed, and reliability offer great challenges [2]. Using terahertz isolators based on magnetic–optical materials is also a possible approach, but their application in waveguide−interconnected terahertz systems is not commonly observed [3,4].

**Figure 1:**
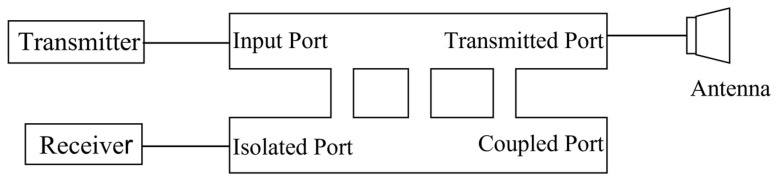
Directional coupler used as a transmit/receive isolation device in THz systems.

Directional couplers, which are extensively utilized in submillimeter and terahertz systems in functions of power isolation, signal mixing, and separation, offer a promising solution. Leveraging the remarkable isolation properties between certain ports, directional couplers are suitable for being transmit/receive isolation devices for terahertz transceiver systems. Consequently, investigating high−isolation directional couplers operating in the THz band holds significant potential for various applications, including miniature terahertz radar and experimental configurations characterized by frequency comb emission [5,6,7,8]. Figure 1 shows the application of a directional coupler in this paper as a transmit/receive isolation device in a terahertz transceiver system.

Many types of terahertz directional couplers have been presented, such as fiber couplers [9], Si dielectric waveguide couplers [10,11], and porous waveguide directional couplers [12,13], etc. Among these, the branch−line waveguide couplers offer considerable advantages in high−frequency applications due to their simple structure, high isolation, and low insertion loss. In 1958, Reed presented the classical design of microstrip branch−line directional couplers, which provided the basic theory of multi−branch waveguide couplers [14]. Nevertheless, since the size of the circuit decreases sharply in the THz band, traditional microstrip line couplers pose processing challenges. Previous studies have demonstrated the effectiveness of computer numerical control (CNC) milling technology in the fabrication of couplers operating in submillimeter bands [15,16,17,18,19]. However, when dealing with components working on a higher frequency which necessitate extremely small branch−line structures, we must employ more costly and time−consuming processing technologies, such as Micro−Electro−Mechanical System (MEMS) or Deep Reactive Ion Etching (DRIE) [20,21,22].

This paper presents two types of 510 GHz branch waveguide couplers, which can be classified as terahertz waveguide couplers. One is a five−branch waveguide coupler structure. Simulation results show that it has good performance but is relatively difficult to manufacture. Therefore, we have redesigned and optimized a new type of three−branch waveguide coupler. Compared to couplers with more branches, it has lower processing difficulty and similar performance. We fabricated and measured both couplers. The design process and simulation result will be detailed in Section 2. Section 3 will cover the fabrication and measurement of the two directional couplers. In the Section 4, we summarize the work presented in this paper, discuss the current limitations, and suggest possible directions for improvement. The design concept and examples of high−isolation terahertz directional couplers provided in the study can be beneficial for designing couplers at higher frequencies and enhancing the performance and reliability of terahertz transceiver systems.

## 2. Design and Simulation

**Figure 2:**
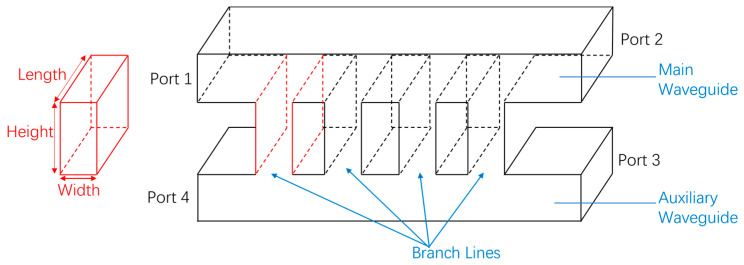
Schematic of a four−branch directional coupler.

Figure 2 depicts a schematic structural diagram of a multi−branch waveguide coupler. The conventional multi−branch waveguide coupler comprises four ports: the input port (Port 1), the through port (Port 2), the coupling port (Port 3), and the isolation port (Port 4). Fed from Port 1, the input signal is split into two parts. One portion reaches Port 2 through the main transmission waveguide, while the other is directed by the branch waveguide to the auxiliary waveguide to reach Port 3. Port 4 remains isolated. According to the research in [23], the parameters of each branch waveguide can be expressed as follows:

*S*_31_ = [(h_1_ + h_2_+ … + h_n_)/λ]^k^, (1)

where n is the number of the shunt branches of the coupler, k is a matching constant independent of the frequency, and λ represents the wavelength of the transmission wave of the designed coupler. For a 3 dB coupler with a center frequency of 510 GHz, the $S_{31}$ is about 0.707 and λ is 0.588 mm. The total width is calculated as 0.465 mm.

Generally, the process for CNC machining of a directional coupler is as follows: The coupler structure is divided into two symmetrical parts by a line extending from the midpoint of the long edge of the main and auxiliary waveguide ports. The corresponding structure is then milled into two metal blocks, which are subsequently joined together. Therefore, the dimension that poses the greatest machining challenge in the entire structure is typically the width of the branch waveguides, which must be larger than the diameter of the smallest milling cutter to be successfully machined.

**Figure 3:**
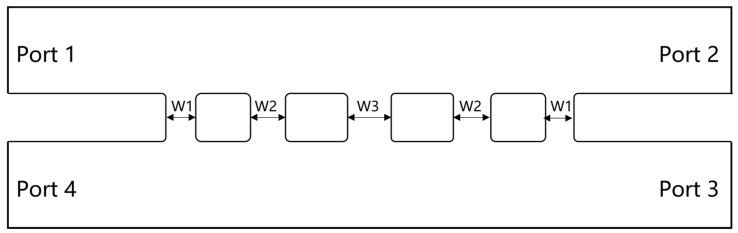
Structure of 510 GHz five−branch waveguide coupler.

**Figure 4:**
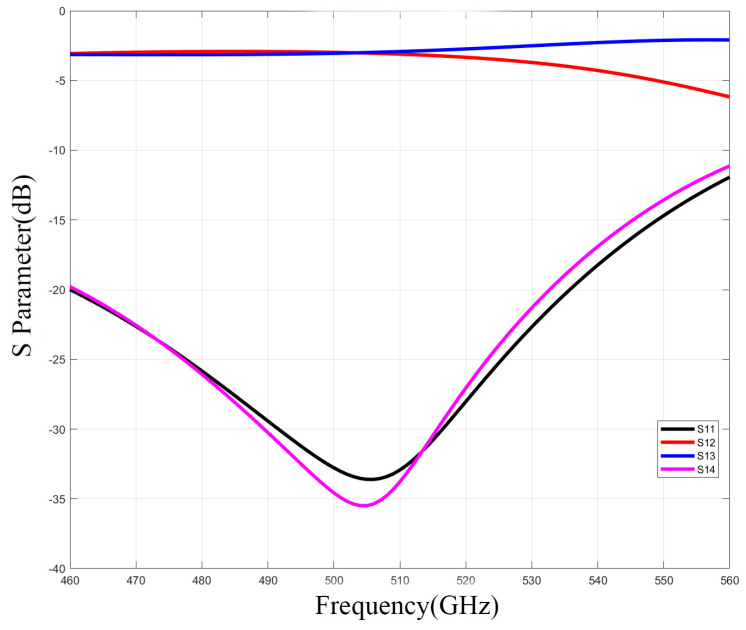
S−parameters simulation of 510 GHz five−branch waveguide coupler.

Typically, incorporating more branches in a waveguide coupler can offer a broader operating bandwidth and increased isolation. However, it can also lead to branches becoming excessively narrow to manufacture, which is particularly important in high−frequency ranges like terahertz. Diminutive dimensions may result in structural fragility and compromised working stability. According to research, [15] and [23], circular cutouts were employed at the terminations of every branch waveguide to enhance performance. Hence, the design proposed in [24] is opted for. The narrowest width of the branch is determined to be 0.08 mm, which could be manufactured by micromachining. Figure 3 illustrates the structure of the five−branch waveguide coupler, and some of the dimensional data are provided in Table 1. We simulated the coupler using the finite element method (FEM) in electromagnetic simulation software. To achieve more accurate results, we set the maximum delta S to 0.002 and the maximum number of passes to 15. The simulation results presented in Figure 4 demonstrate that it achieves isolation exceeding 30 dB at the central operating frequency of 510 GHz.

**Figure 5:**
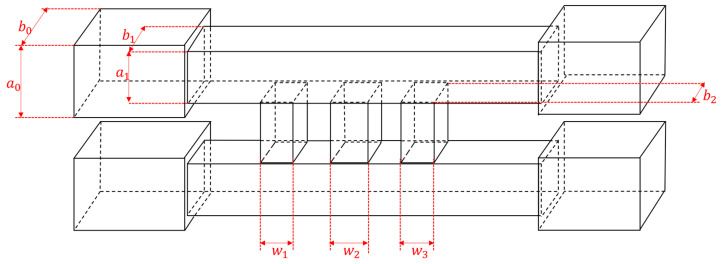
Model of novel 510 GHz three−branch waveguide coupler.

**Figure 6:**
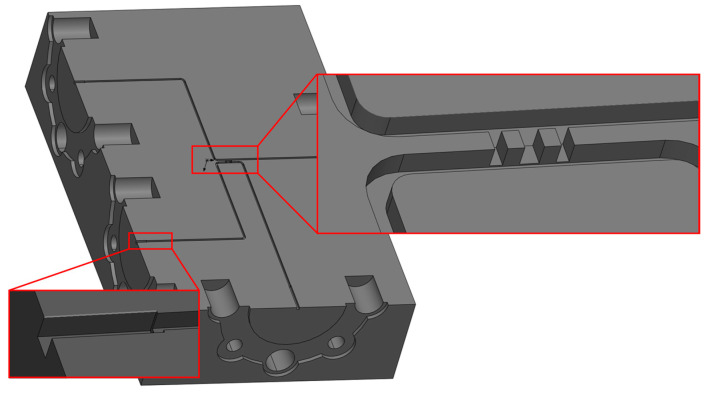
Layout of novel 510 GHz three−branch waveguide coupler.

**Figure 7:**
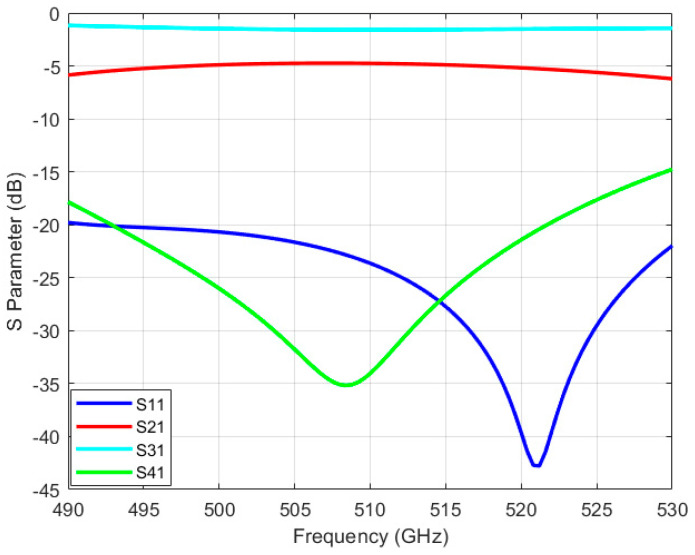
S−parameters simulation of novel 510 GHz three−branch waveguide coupler.

Although the five−branch directional waveguide coupler has good performance, its narrowest branch width is only marginally greater than the minimum diameter of microfabrication milling cutters. Therefore, we aim to reduce the processing difficulty further to ensure successful and efficient manufacturing. The simplest way is to reduce the number of branches, which unfortunately would significantly decrease performance. Thus, we made some improvements to the structure. Firstly, simulations indicate that decreasing the dimensions of both the primary and auxiliary waveguides can shift the operating frequency range of the coupler toward higher frequencies, which means by reducing the size of the main waveguide and the auxiliary waveguide, the size reduction of the branch waveguide can be partially mitigated. For integration with other system components, the main and auxiliary waveguides are initially sized at WR1.9 (0.48 mm × 0.24 mm). Still, they are reduced to 0.38 mm by 0.19 mm within the core structure of the coupler. Secondly, since the minimum size is typically the width of the branch waveguide, we can trade off the length of the branch waveguide for a wider width to ensure that the branch waveguides still retain a similar ability to conduct electromagnetic waves. Figure 5 provides the modal of the modified structure. Compared with the traditional branch waveguide coupler structure shown in Figure 2, it can be seen that the length of the branch waveguide is usually the same as that of the main and auxiliary waveguides. However, in our modified structure, the length of the branch waveguide is slightly shorter than that of the main and auxiliary waveguides, resulting in a larger width. Some important dimensions of the novel three−branch waveguide coupler are given in Table 2, and the layout is shown in Figure 6. With the aforementioned improvements, we increased the narrowest branch width from 80 μm to 110 μm while maintaining high isolation at 510 GHz. Simulation results are illustrated in Figure 7. Isolation at 510 GHz exceeds 34 dB and is maintained over 20 dB within the range of 493–520 GHz.

## 3. Measurement Result

**Figure 8:**
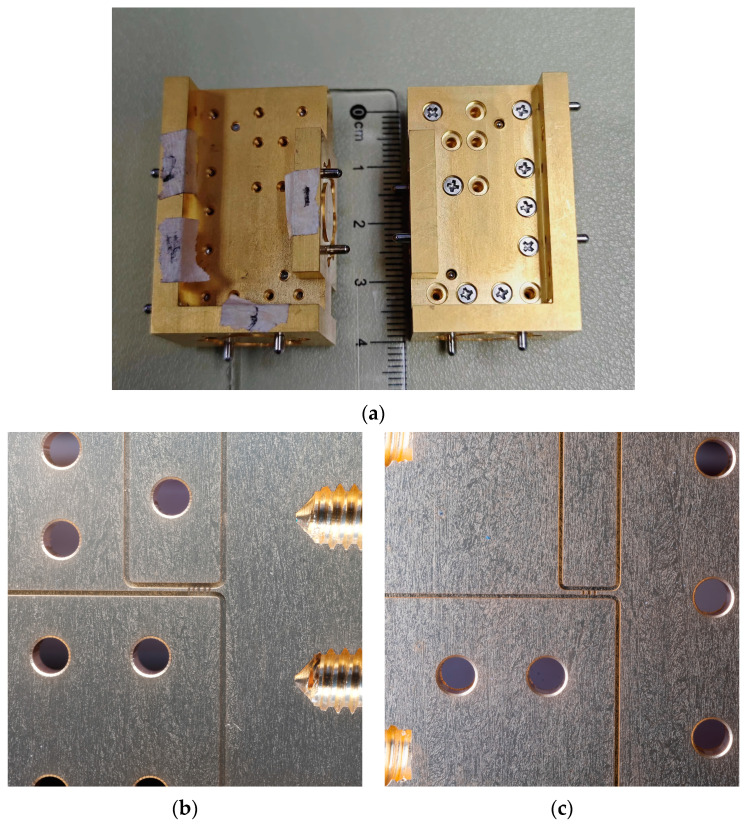
Photographs of fabricated waveguide couplers. (**a**) The two processed waveguide couplers. (**b**) The core structure of the five−branch waveguide coupler. (**c**) The core structure of the novel three−branch waveguide coupler.

**Figure 9:**
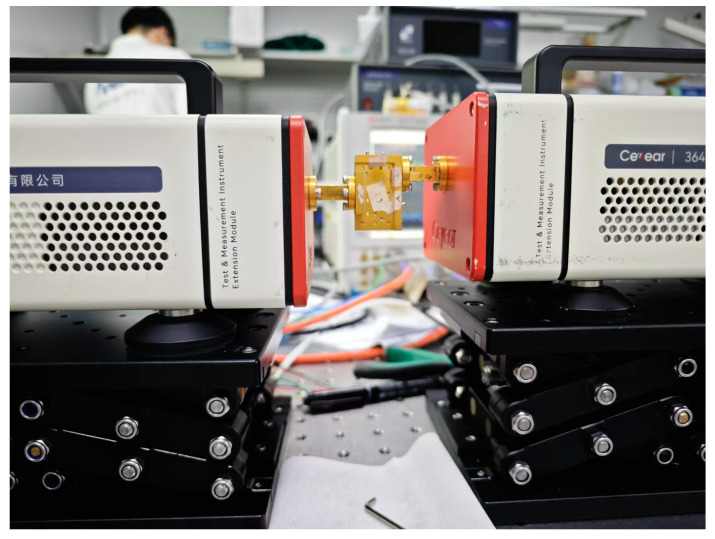
Measurement setup for the couplers.

The two proposed branch waveguide couplers are manufactured into split blocks by CNC milling, with dimensions of 40 mm × 25 mm × 20 mm. Photographs of the fabricated waveguide couplers are given in Figure 8. The S−parameters were measured with a vector network analyzer with extension transmitter and receiver modules, as shown in Figure 9. Due to experimental limitations, only the performance within the 500–540 GHz range was tested. The actual operating bandwidth of the devices should be wider. Due to a waveguide size mismatch between the vector network analyzer’s WR−1.5 extension spectrum module port and the WR−1.9 coupler port, a higher standing wave will be generated in the absence of a transition waveguide. Moreover, mainly limited by additional losses caused by surface roughness, the measured insert loss is larger than the simulation.

**Figure 10:**
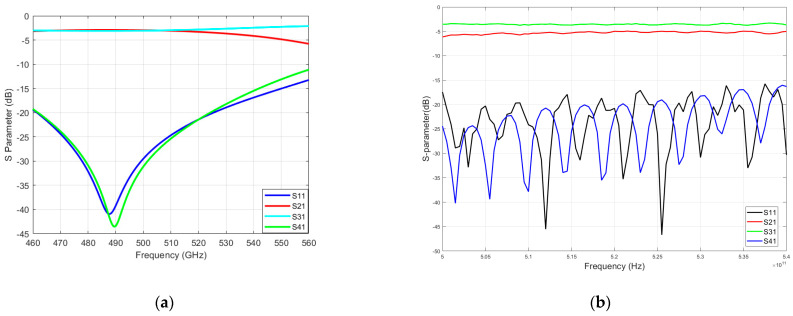

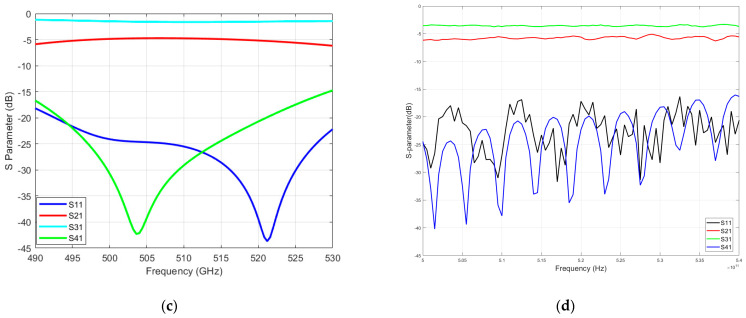
Simulation and measurement S−parameters of waveguide couplers. (**a**) Simulation results for the five−branch waveguide coupler connected to the measurement system. (**b**) Measured S−parameter of the 510 GHz five−branch waveguide coupler. (**c**) Simulation results for the novel three−branch waveguide coupler connected to the measurement system. (**d**) Measured S−parameter of the novel 510 GHz three−branch waveguide coupler.

As previously mentioned, to ensure good connectivity with our transceiver system, the coupler ports use WR1.9 waveguides, while the measurement system uses WR1.5 waveguides. To investigate the impact of this mismatch on the test results, we conducted simulations using electromagnetic simulation software. Figure 10 illustrates the simulation results and actual test results of two couplers when directly connected to a WR1.5 vector network analyzer. We found that mismatches of waveguides between the measurement system and the device caused the frequency point of minimum isolation to shift to the left. This explains why the measured isolation performance is inferior to the simulated results. With a range of 500–540 GHz, both couplers have a return loss and isolation of more than 15 dB. Because of the port standing wave and possible weak connection, the measured isolation curve is serrated. In our system, we pay special attention to the performance of isolation at the center frequency point, so both couplers meet the requirement of a transmit/receive isolation device in our terahertz system.

In Table 3, we provide a thorough comparison of our designs with prior works operating at similar frequencies. Our designs achieve enhanced isolation in the proximity of the center frequency while exhibiting reduced machining complexity, which makes them more suitable to be used as transmit/receive isolation devices in terahertz transceiver systems.

## 4. Discussion and Conclusions

In this paper, to meet the demands of transmit/receive isolation devices within terahertz transceiver systems, two types of branch waveguide couplers working in terahertz bands are presented. One is the five−branch waveguide coupler, which primarily adopts the classic multi−branch coupler structure. The five−branch waveguide coupler exhibits commendable performance, but the microfabrication is challenging relatively. Although it can still be fabricated using standard CNC technology, which is a very well−used and cost−effective method, the minimum dimensions are nearly at the limit of what this process can achieve. As the operating frequency increases further, this design may no longer be applicable. Therefore, a novel three−branch waveguide coupler was devised to alleviate processing complexities while maintaining high isolation around the center frequency. By reducing the size of the main and auxiliary waveguides and shortening the branch waveguides, structural improvements have increased the minimum dimensions of the coupler, making it easier to manufacture using CNC technology. Although the performance may slightly decline, this approach still offers a viable design strategy for directional couplers with branch waveguides at higher frequencies. When performance requirements are less stringent but low−cost manufacturing methods such as CNC machining are desired, our design can serve as a useful reference. Both couplers have been fabricated and measured, confirming that they offer higher isolation near the center frequency.

Certainly, there are some limitations to this work. Since our design’s primary objective was to meet the isolation requirements of practical terahertz transceiver systems, we are more concerned with the isolation at the center frequency point and how much lower the isolation is compared to the transmission losses of the signal and the echo through the coupler, as this determines the system’s signal−to−noise ratio. Therefore, parameters such as amplitude balance and phase balance were overlooked. The designs are not standard 3 dB couplers, which somewhat limit their applicability. This is due to the specific parameter settings we employed. However, we believe that when addressing different requirements, our design can still be referenced. By adjusting the dimensions, it is possible to develop a coupler that better balances various performance parameters. Additionally, due to the mismatch between the test ports and the device ports, we were unable to assess the device’s performance accurately. In the future, we need to find better testing methods to address this issue.

In summary, we present the design, fabrication, and testing of two directional multi–branch couplers with practical engineering value. Both directional couplers have a low manufacturing cost and high working frequency. This not only meets the engineering requirements of our system but also inspires the design of directional couplers for higher frequencies under the constraints of processing technology.

## Figures and Tables

**Table 1:** Size of 510 GHz five−branch waveguide coupler.

Edge	*w*_1_	*w*_2_	*w*_3_
Length/mm	0.08	0.1	0.12

**Table 2:** Size of novel 510 GHz three−branch waveguide coupler.

Edge	*a*_0_	*a*_1_	*b*_0_	*b*_1_	*b*_2_	*w*_1_	*w*_2_	*w*_3_
Length/mm	0.24	0.48	0.19	0.38	0.35	0.11	0.14	0.12

**Table 3:** Performance of different directional couplers.

Ref.	Working Frequency (GHz)	Isolation (dB)	Narrowest Branch Width (um)	Processing Method
[15]	380–460	15	90	CNC
[16]	380–500	22	70	CNC
[20]	350–410	20	60	DRIE
[21]	335–415	20	45	UV−LIGA
[19]	282	15	192	CNC
Five−branch waveguide coupler in this work	510	35	80	CNC
Three−branch waveguide coupler in this work	510	35	110	CNC

## Data Availability

The data that support the findings of this study are available from the corresponding author upon reasonable request.
